# Exploring the gender care experiences and perspectives of individuals who discontinued their transition or detransitioned in Canada

**DOI:** 10.1371/journal.pone.0293868

**Published:** 2023-11-29

**Authors:** Kinnon R. MacKinnon, Wren Ariel Gould, Gabriel Enxuga, Hannah Kia, Alex Abramovich, June S. H. Lam, Lori E. Ross

**Affiliations:** 1 School of Social Work, York University, Toronto, Ontario, Canada; 2 Dalla Lana School of Public Health, University of Toronto, Toronto, Ontario, Canada; 3 School of Social Work, The University of British Columbia, Vancouver, British Columbia, Canada; 4 Institute for Mental Health Policy Research, Centre for Addiction and Mental Health, Toronto, Ontario, Canada; 5 Adult Gender Identity Clinic, Centre for Addiction and Mental Health, Toronto, Ontario, Canada; Northumbria University, UNITED KINGDOM

## Abstract

**Background:**

Those who detransition have received increased public and scholarly attention and their narratives are often presented as evidence of limitations with contemporary gender-affirming care practices. However, there are scant empirical studies about how this population experienced their own process of gaining access to gender-affirming medical/surgical interventions, or their recommendations for care practice.

**Aims:**

To qualitatively explore the care experiences and perspectives of individuals who discontinued or reversed their gender transitions (referred to as *detransition*).

**Methods:**

Between October 2021-January 2022, Canadian residents aged 18 and older with experience of stopping, shifting, or reversing a gender transition were invited to participate in semi-structured, one-on-one, virtual interviews. A purposive sample of 28 was recruited by circulating study adverts over social media, to clinicians in six urban centres, and within participants’ social networks. Interviews ranged between 50–90 minutes, were audio-recorded, and transcribed verbatim. Following constructivist grounded theory methodology, interview data were analyzed inductively and thematically following a two-phase coding process to interpret participants’ experiences of, and recommendations for, gender care.

**Results:**

Participants were between the ages of 20–53 (71% were between 20–29). All participants identified along the LGBTQ2S+ spectrum. Twenty-seven out of 28 of the participants received medical/surgical interventions (60% were ages 24 and younger). A majority (57%) reported three or more past gender identities, with 60% shifting from a binary transgender identity at the time of initiating transition to a nonbinary identity later in their transition journey. To access medical/surgical interventions, most participants were assessed via the gender-affirming care model pathway and also engaged in talk therapy with a mental healthcare provider such as a psychologist or psychiatrist. Some participants experienced their care as lacking the opportunity to clarify their individual treatment needs prior to undergoing medical/surgical transition. Decisional regret emerged as a theme alongside dissatisfaction with providers’ “informed consent” procedures, such that participants felt they would have benefitted from a more robust discussion of risks/benefits of interventions prior to treatment decision-making. Overall, participants recommended an individualized approach to care that is inclusive of mental healthcare supports.

**Conclusions:**

To optimize the experiences of people seeking and receiving gender care, a thorough informed consent process inclusive of individualized care options is recommended, as outlined by the *World Professional Association of Transgender Health*, *standards of care*, *version 8*.

## Introduction

A recent Pew research poll found that roughly five percent of Americans under age 30 identify with a gender different than their assigned sex at birth [1]. In Canada, there are over 100,000 transgender, nonbinary, and gender-diverse people (TGD), comprising approximately 0.33% of the national population [2]. Some TGD people seek transition-related gender care such as hormonal or surgical interventions to align their sexual characteristics with their gender identity and/or to alleviate Gender Dysphoria—a diagnosis listed in the *Diagnostic and Statistical Manual of Mental Disorders* (DSM) [3,4]. In the past ten years, referrals to gender identity services show a marked rise in the United States, in Canada, and globally [4–8].

In recent years some clinicians and researchers have expressed concerns about predominantly young TGD individuals assigned female at birth (AFAB) who now comprise a majority of adolescent gender care service referrals in high-income nations, igniting debates about how to best clinically support this population [9]. This group is thought by some to present a particular risk of decisional regret or of detransition [10,11]. In this article, we conceptualize detransition as stopping, shifting, or reversing a gender transition in connection with a change in the individual’s self-conceptualized gender identity [12–15]. Current data estimating the prevalence of detransition associated with a change in gender identity are lacking due to methodological limitations. However, recent studies with clinical samples have found that between 8.1%-9.8% of children, adolescents, and young adults may detransition to their birth-assigned gender or to nonbinary after initially being assessed and referred for gender care medical interventions and/or medically transitioning [16–18]. Those who reverse a gender transition—some of whom self-label as detransitioners or detrans—have been presented as reason to safeguard adolescents from hasty medical decisions [11,18,19].

Emerging exploratory studies shed insights into the care seeking experiences of those who subsequently detransitioned and/or who expressed decisional regrets. Vandenbussche’s survey of 237 detransitioners (92% AFAB; 65% of whom medically transitioned/detransitioned) found that 70% reported they detransitioned because they realized their gender dysphoria was related to other factors [13]. Forty-five percent felt under-informed about the health consequences of transition-related medical care before undergoing treatment, and 60% reported feelings of regret. Littman’s survey of 100 individuals (69% AFAB) who detransitioned following medical/surgical interventions found that 55% felt they did not receive an adequate medical/mental health assessment prior to initiating care and 38% felt past experiences of trauma or poor mental health may have contributed to their feelings of gender dysphoria [15]. In both of these studies, “internalized homophobia”, or what may have been previously defined as ego-dystonic homosexuality [20] was another explanation for participants’ desire for transition and eventual detransition. Pullen Sansfacon et al.’s qualitative investigation into 20 detrans youth’s (95% AFAB) transition and detransition journeys discovered a theme of some participants misattributing their feelings to an experience of gender dysphoria or of being transgender [21]. Twelve participants experienced negative feelings such as grief, regret, or anger about the initial transition, with participants also reporting positive feelings such as self-discovery. MacKinnon et al.’s qualitative examination of detransition-related healthcare experiences among 28 individuals (64% AFAB) identified a range of factors for detransitioning such as physical or mental health decline during transition, shifts in gender identity, and transgender discrimination [14]. A majority were satisfied with gender care interventions, with one-third of the sample reporting decisional regret or ambivalent feelings about their past medical/surgical care [14].

Applying a qualitative, interview-based study design, this article presents the gender care seeking and receiving experiences of individuals living in Canada who eventually discontinued their transition or detransitioned. We first review three major models of gender care: 1) the gender-affirming model of care; 2) the informed consent model; and 3) the gender exploratory therapy model. We then present our methods, findings, and a discussion about implications for care practice and research.

### The gender-affirming model of care

The primary philosophy underpinning the gender-affirmative model of care is that gender presentations can vary across cultures and that no gender identities are pathological [22,23]. Proponents of the gender-affirmative model of care encourage a curious clinical position, recommend fostering a neutral space for gender exploration without favouring any particular outcome, while also respecting an individual’s affirmed name, pronoun, and gender identity [23–25]. The *World Professional Association for Transgender Health Standards of Care*, version 7 (*SOC-7*), recommends for TGD adult care-seekers one professional assessment be completed prior to the initiation of gender-affirming hormones or chest/breast surgeries, and two for lower/genital surgeries.^4^ Assessments are completed by providers who are competent with the DSM and able to conduct an “assessment of gender identity and gender dysphoria, history and development of gender dysphoric feelings” and determine whether any mental diagnoses are present and “well-controlled” prior to initiating hormones/surgeries (p. 23) [4]. In practice, this means that a person seeking these interventions must undergo psychosocial assessments which may include discussions about social/familial supports, financial and housing considerations for surgeries and surgical aftercare, experience living in one’s felt gender, as well as an evaluation to assess mental health/illness [26]. In Canada, wait-times to access an assessment for pediatric, adolescent, or adult transition-related gender care can vary between a few months to over one year [27,28].

Opponents of the gender-affirmative model of care have argued that affirming young people’s TGD identities, in particular, may unduly encourage the pursuit of medical/surgical interventions that could be later regretted [10,29]. The updated SOC version 8 (*SOC-8*), released September 15, 2022, recommends a comprehensive assessment for adolescents seeking medical/surgical interventions, and that adolescents should be offered the opportunity to explore their gender without favouring any identity outcome [30]. For adults, the *SOC-8* recommends a comprehensive assessment for only complex cases or for those who are requesting treatments with limited research evidence, and that assessors should be able to distinguish gender dysphoria from co-existing mental or psychosocial issues [30]. Our study was conducted prior to the release of the *SOC-8*.

### The informed consent model

The informed consent model (ICM) does not require an assessment of gender identity or gender dysphoria prior to the initiation of gender-affirming hormones [31,32]. Assuming a gender-affirming position, the ICM proposes that TGD people who understand the risks and benefits of gender-affirming hormones can initiate treatment without a diagnosis of gender dysphoria and/or gender assessment [33,34]. The ICM emerged as an alternative to the *SOC*, to depathologize trans experiences [32,35], and to reduce barriers to care [36,37]. ICMs are heterogeneous, lying on a continuum ranging from “weak” (e.g., some emphasis on assessment from a perspective of collaboration and patient-led exploration of gender dysphoria) to “strong” (e.g., no assessment of gender dysphoria, while bolstering the informed consent process and patient education surrounding treatment options to meet individual embodied gender goals) [38]. The ICM may be reflective of broader trends in healthcare which move away from paternalism and toward patient collaboration, while centering the ethical principles of autonomy and self-determination—such as “relationship-centered care” [34]. A post-intervention survey administered to adult patients (mean age: 25, range 21–30) seeking gender-affirming hormones found that the ICM is associated with high patient satisfaction [39].

Still, scholars, care providers, and the authors of the most recent *SOC-8* [30] have suggested that while the gender-affirming model of care can be complementary to the ICM, for some adolescents, identity fluidity may be developmentally-typical such that the ICM may not be appropriate. For example, the *SOC-8* states that there are “no studies of the long-term outcomes of gender-related medical treatments for youth who have not undergone a comprehensive assessment… [this approach] carries the risk that the decision to start gender-affirming medical interventions may not be in the long-term best interest of the young person at that time” (p. S51) [30]. A Canadian prospective cohort study comprising 174 TGD youth (79% AFAB) selected from 10 pediatric gender clinics found that five of these clinics do not require a psychiatric or psychological assessment prior to initiating hormone blockers or gender-affirming hormones [40].

### The gender exploratory therapy model

The gender exploratory therapy model is characterized by required, in-depth talk therapy over an extended period of time in an attempt to foster an understanding of underlying factors that may be contributing to gender-related distress [41]. Proponents argue that this approach is developmentally-informed while also enabling clinical discrimination between gender dysphoria and mental illness prior to facilitating medical/surgical interventions [41,42]. Practitioners of gender exploratory therapy also often view medical transition as a last resort availed to patients only after judicious therapy and evaluation [42]. To date, known practitioners of this model primarily work clinically with children and adolescents [43], however Spiliadis [42] states that gender dysphoric children, adolescents, and adults alike could benefit from gender explorative therapy.

Clinicians who promote the gender exploratory therapy model have raised concerns regarding both the gender-affirming care model and the ICM due to their “gender-affirmative” stance and for assuming that medical/surgical interventions alone can ameliorate the young person’s distress [41]. Marchiano states “therapists are contributing to the rise in transitioning by quickly validating a young persons’ self-diagnosis as transgender without careful differential diagnosis, exploration of trauma, questions about sexual orientation, etc” (p.357) [29]. She further states that some young detransitioned women—only after medical transition—come to understand themselves as gender non-conforming sexual minorities (lesbian, bisexual, or queer), without having been provided an opportunity to therapeutically explore their life histories as they relate to sexuality, trauma, and feelings of gender dysphoria [29].

To date, there are no known empirical studies that examine psychosocial or medical/surgical outcomes following the gender exploratory model of care and some authors have raised questions regarding its clinical practices. For instance, this model has been likened to gender identity conversion therapy given that some practitioners avoid using clients’ affirmed name and pronouns, while aiming to question trans identification in children and adolescents [44]. However, Spiliadis’ differentiates the exploratory model from identity conversion therapy, stating that practitioners of the former are advised to acknowledge patients’ gender identities, and to collaborate with them without any active guidance toward a specific identity or outcome [42]. Other authors have argued that by not providing an estimated length of time for explorative talk therapy, this approach raises ethical tensions given that delaying medical interventions may compound mental suffering in TGD young people [45]. Finally, it is important to note that many who promote the gender-affirming model of care do encourage gender exploration as a means of supporting a young person’s self-understanding and informed decision-making prior to making medical decisions [44,46].

## Materials and methods

Our study sought to understand how participants perceived their own process of initiating gender care, including their interactions with care providers and the delivery of care, how they felt about their decision to transition, and their insights into how care provision might be improved. We applied an exploratory, qualitative study design—constructivist grounded theory (CGT)—that seeks to uncover the ways in which participants make meaning from social processes and within relations of power, and to co-develop empirical knowledge from participants’ lived experience [47,48]. Charmaz suggests that CGT can offer valuable insight on phenomena that are underexplored, disregarded, or poorly understood [47]. Ethics approval was obtained through the York University Research Ethics Board and all participants provided verbal consent to participate. To enhance rigour and community engagement in our study design, two individuals with experiences of detransition were consulted in the development of the study recruitment flyer and the interview guide. Our study team includes TGD and cisgender researchers, including individuals who have detransitioned.

### Recruitment and data collection

To recruit participants, we circulated study flyers over social media, to private internet-based detransition support groups, and to clinicians in six urban centres across Canada. Adverts were also distributed to health centers and community organizations serving lesbian, gay, bisexual, trans, queer, and two-spirit (LGBTQ2S+) people in Canada. Following purposive and snowball sampling methods, participants with experience of reversing their gender transition and/or re-identifying with their birth-assigned sex were particularly encouraged to share study advertisements within their personal networks of detransitioners. To achieve greater demographic diversity in the sample, flyers specifically inviting individuals who were assigned male at birth (AMAB) and Black, Indigenous, and other people of colour (BIPOC) were also circulated. Participants who expressed interest in the study were screened to confirm they met eligibility criteria before being invited to participate. Eligibility criteria included: aged 18 and older, living in Canada, ability to participate in an interview in one of Canada’s two official languages–English or French–and self-identifying as detransitioning, retransitioning, detrans, retrans, re-identifying, experiencing a shift in gender identity after transitioning, or having stopped a gender transition. Gender transition was defined as inclusive of social, legal, and/or medical transition.

Participant recruitment and data collection occurred between October 2021 and January 2022. Data were collected in the form of in-depth, semi-structured, one-on-one virtual interviews that ranged from 50 to 90 minutes. A total of 28 participants met eligibility criteria and fully completed virtual interviews via Zoom, as well as a demographic questionnaire. Participants received a $30 CAD gift card for participating. Twenty-six interviews were conducted in English by either KM or GE and two were conducted in French by FA. We directly asked participants the following questions, among others: “To affirm a trans or nonbinary identity, what steps did you take to transition the first time?; Did you see a healthcare professional like a doctor, social worker, or psychologist?; What type of pathway did you go through to access gender-affirming healthcare?; At the time, how did you feel about physically transitioning (including taking hormones, having surgeries, or other physical interventions such as hair removal)?”. We also asked follow-up probing questions to gather more information relating to participants’ overall process of initiating gender care. To understand how participants felt about their decision to transition, we asked the question: “Looking back, do you feel like transitioning was the right or wrong decision for you at the time?” which offered them space to reflect and discuss feelings of decisional regret and/or satisfaction. Finally, participants were asked: “What are your main recommendations for care providers who want to provide better care and support people who are de/retransitioning?” which elicited rich feedback regarding their perspectives about gender care delivery.

### Data analysis

We specifically analyzed narratives of seeking and receiving gender care, feelings of satisfaction or dissatisfaction with medical/surgical care interventions, and recommendations to improve care delivery. From these in-depth accounts we were able to discern the model of care each participant had experienced in the process of accessing interventions and how they felt about this clinical approach. We further analyzed narrative data to determine the extent to which participants felt decisional regret and/or undesirable outcomes with hormonal/surgical interventions to contextualize their overall perspectives on the care they received. When dissatisfaction with interventions or decisional regret emerged in the interview data, we closely analyzed these transcripts to understand how these participants described their transition journey and the care they received.

Our data collection and analysis process was iterative and inductive, following two primary analytic steps. First, upon completion of the first 20 interviews, KM and GE reviewed these transcripts and developed an initial draft coding framework which conceptualized preliminary findings thematically. In the second step of data collection and analysis, the final eight interviews were conducted, and these transcripts reviewed, to confirm data saturation. As we reached data saturation across the interviews, we finalized the thematic coding framework. All transcripts were read, re-read, and coded by a minimum of three authors (KM, GE, and WAG). Involving three project team members in reviewing transcripts and developing the coding framework contributed to study rigor by enabling the assessment of data saturation and open discussion among coders to consistently and reflexively interpret overarching themes from the interview data. Dedoose analytic software was also used to support systematic development of the coding framework, and to code the transcripts. As such, we followed a reflexive and inductive coding process to develop themes grounded in the data, as described by Charmaz who developed CGT methods [47,48]. During data analysis, we consulted with two Canadian psychiatrists who work in gender care and several detransitioners. Following data analysis, we contacted all study participants with an update regarding plans to share their anonymized narrative data over social media, to presentations to care providers, and in academic writing. Participants were provided the option to request for their anonymous narratives to not be shared, and one participant opted out due to privacy concerns, but did not withdraw from the study. For further description of our methods, please see [14].

## Results

Among the 28 participants, 18 (64%) were AFAB and 10 (36%) were AMAB. A majority (57%) reported three or more past gender identities and 11 initiated social gender transition under the age of 18. Several participants who affirmed a trans/nonbinary identity at the time of interview indicated a history of detransition in which they returned to expressing their gender in alignment with their assigned gender, followed by retransitioning to re-identify as TGD. Twenty-seven initiated gender-affirming hormones and, of these, 11 also underwent surgeries inclusive of one or more of the following: double mastectomy (n = 8); breast augmentation (n = 1); gonadectomy (n = 6); genital surgery (n = 3). One participant reported use of puberty blockers prior to testosterone therapy. One participant sought to partially undo metoidioplasty, to the extent possible, which is reported elsewhere [14]. Participants largely resided in Canada during transition/detransition/retransition, with the exception of one person who initiated hormones at an “informed consent” clinic in the US. See Table 1 for more information regarding participant demographics, Table 2 for a presentation of participants’ reports of satisfaction/dissatisfaction/regret with interventions, and Table 3 for illustrative quotes that support the thematic analysis.

**Table 1:** Participant demographic characteristics.

Participants (n = 28)	No.	%
***Birth-assigned sex***		
Assigned female at birth	18	64%
Assigned male at birth	10	36%
***Age***		
20–29	20	71.4%
30–39	6	21.4%
40+	2	7.1%
***Age of social transition***		
<15	5	17.9%
15–17	6	21.4%
18–24	12	42.9%
25–29	3	10.7%
30+	2	7.1%
***Age of medical transition***		
15	2	7.1%
18–24	15	53.6%
25–29	6	21.4%
30+	4	14.3%
Not applicable	1	3.6%
***Age of detransition***		
<18	1	3.6%
18–24	13	46.4%
25–29	9	32.1%
30+	5	17.9%
***Current sex/gender identity***		
Female/Woman	6	21.4%
Nonbinary	5	17.9%
Nonbinary female	1	3.6%
Nonbinary & gender-fluid	3	10.7%
Nonbinary, gender-fluid male	1	3.6%
Nonbinary male	1	3.6%
Trans & nonbinary	6	21.4%
Trans	2	7.1%
Undecided/Questioning	3	10.7%
***Number of past*** ***gender identities***		
Two	12	42.9%
Three	6	21.4%
Four	4	14.3%
Five	4	14.3%
Six	0	0%
Seven	2	7.1%
***Sexual Orientation***		
Asexual	1	3.6%
Bisexual/pansexual	9	32.1%
Gay/lesbian/homosexual	10	35.8%
Heterosexual	1	3.6%
Queer	7	25.0%
***Race/Ethnicity***		
Jewish (White)	2	7.1%
Multi-racial (includes Black, Indigenous, Arab, Latinx, & South Asian)	5	17.9%
White	21	75.0%
***Self-reported mental health/neurodiversity***		
Autism Spectrum	5	17.9%
Borderline Personality Disorder (BPD)	4	14.3%
Attention Deficit Hyperactivity Disorder (ADHD)	3	10.7%
Neurodivergent (general)	2	7.1%
Depression	2	7.1%
Anxiety	1	3.6%
Bipolar Disorder	2	7.1%
Schizophrenia	1	3.6%
Post-Traumatic Stress Disorder (PTSD)	1	3.6%
Obsessive Compulsive Disorder	1	3.6%
Traumatic Brain Injury	1	3.6%
**Self-reported disability (including mental health)**		
Yes	16	57.1%
No	12	42.9%

**Table 2:** Participant reports of satisfaction/dissatisfaction/regret.

*Intervention*	*Dissatisfaction/* *Complications*	*Satisfaction/* *No regret*	*External regret*	*Internal regret*
**Puberty Blockade (n = 1)**	1	0	1	0
**Gender-affirming hormones (n = 27)**	12	15	5	6
**Chest masculinization, double mastectomy** **(N = 8)**	0	5	2	2
**Breast augmentation (n = 1)**	0	0	1	1
**Gonadectomy (n = 6)**	3	1	3	3
**Genital surgery (n = 3)**	1	2	1	1

**Notes:** A majority of participants reported satisfaction, and no decisional regret, with gender-affirming hormones/surgeries. *Dissatisfaction* indicates medical/surgical complications or undesirable outcomes with an intervention. Twelve participants discussed dissatisfaction/complications with hormonal treatments. *External regret* refers to decisional regret emerging from feelings of external pressure on the individual’s transition, and/or perceived care system/provider failures. *Internal regret* refers to decisional regret emerging from within the individual, such as a change in identity or feeling of having made the wrong decision in the past. External and internal regret often co-occurred.

**Table 3:** Illustrative quotes.

Theme	Illustrative Quotes
***Binary Gender Assessments and Linear Transitions***	
	I only remember the [assessment] question about clothes and do I “wear the clothes of the other gender?” because that just stuck out in my mind as a silly thing to ask. But I don’t remember, I think she had two or three other questions but I don’t really remember what they were. I remember being a little unimpressed by the questionnaire. But I don’t know if she was going through some DSM criteria or what she was doing (Participant 25, nonbinary, age 28).
	It was a long and hard year of waiting and getting nowhere. So what ended up happening was, [doctor] tested me for all the things he could think of, and never had any concrete results, so he referred me to an endocrinologist. But then the wait for the endo was months, and I was just like, “Look, I literally can’t wait any longer; it’s been two years at this point and especially this past year has been really hard.”… I was on testosterone for two and a half years, and decided to stop around the time that I came out as nonbinary. (Participant #3, nonbinary, age 23).
	Nonbinary identities and transition wasn’t really something that we had a whole lot of information about. I was so scared about having my experience invalidated, not being able to get [chest] surgery, or not being able to have the option for testosterone, because everything was pushed through that binary lens. I still felt like I was pushed and hurried into a category… I said what I said to jump through the hoops to be able to get to what I thought I needed, and as it turns out, some of those interventions, the hormones, just didn’t work for me in the way that it did for everyone else. Maybe I wouldn’t have been so afraid that I wouldn’t be able to get top surgery unless I proved that I was binary, trans and needing that… just to get through the red tape for the sake of healthcare professionals or psychiatrists or getting those letters (Participant #6, transmasculine nonbinary, age 36).
	I think my first couple of appointments [at the trans clinic] were like… first getting to know me… asking me questions about like, childhood and why I felt like I was trans. I think the third appointment they were talking more about hormones, what you can expect like as far as changes go. And then I think they also ended up doing bloodwork around that time. Just to get enough time for the results to come back… And then I think it was by the time I got to like my sixth appointment was when they were like “OK. Make another appointment with us and we can start you on hormones”… And because of [BPD] they also wanted a note from my therapist saying that you know, I’m OK and [mentally] healthy enough to have this treatment… if you have a diagnosis of BPD, they are very kind of wary about like starting hormones. Unless you’ve been treated (Participant #20, female, age 25).
	Before 2019 we had no [gender-affirming surgery] coverage at all. Unless you wanted to fly out of the province and have a mental health assessment, and I’ve been told that the assessment was really bad. And they could deny you over things like your sexuality. So, I just didn’t apply… We can’t access [surgery] in province, and I’ve been on a waitlist ever since we got the partial [insurance] coverage three years ago… I’m kind of at a breaking point where I’m just considering detransitioning because it’s just so awful here essentially… You can’t meaningfully transition here… [These policies] put me in a situation where I don’t even know my identity anymore (Participant #27, undecided, age 25).
***Dissatisfaction with*** ***“Informed Consent” Practices*** ***and Decisional Regrets***	
	I’m still happy with the fact that I got top surgery, but… you know, the hormones just weren’t meant for me in the same way… For me, my voice didn’t really drop all that much, that was a big one for me [voice] dysphoria was big… a lot of non-binary folks are getting lost, they’re falling through the cracks in the system. (Participant #6, transmasculine, nonbinary, age 36).
	I might have had some naïve ideas about what gender transition was going to be like… So like the stuff I was seeing was–the transition timelines that people post to their social media or you see the before and after pics, yeah. So I’d see these trans women and they’d be like, “I’m one year on HRT, look how good I look.” And I’d be like, “holy crap, HRT does all that?” And I’m like, OK half of that was not the HRT, that was lighting and makeup. So I think I just didn’t really fully appreciate what [transition] would be like… I’ve had at various points feelings of regret about transition, both in terms of regretting going on hormones or regretting how soon I decided to go on hormones. Sometimes I think maybe I should have explored my identity a bit before I just jumped into that. And I sometimes have regret about even just my name and stuff and the fact that I’ve changed my legal gender. (Participant #25, nonbinary, age 28)
	In terms of trans culture, like [when I was living as a trans woman] I got really excited about possible breast development. But then it turned out that my body wasn’t going to grow the breasts I imagined. (Participant #17, gender-fluid male, age 32).
	I think what hurt, what made me feel frustrated was after I had my double mastectomy… my mental health started spiraling pretty quick… I really had this expectation that transition was just–everything was going to be better. Like I was just going to be this new person and I–everything was going to be fine and it just wasn’t. And I think what hurt was [my doctor] had sent me to the hospital to get a [psychiatric] assessment done and I got diagnosed with BPD and PTSD. I felt a little frustrated because I didn’t understand why this stuff was getting diagnosed after and not before (Participant #22, female, age 29).
	When I went to therapy, she basically was like “Sounds like you’re a man.” …And she had told me in the first 10 minutes that she’ll send a letter to my GP [family physician] saying that I’ll benefit from transition, before we even really spoke. And she had to see me for three sessions because, I’m fairly certain, is the law, in [province]. [After detransition] I started talking about it with my psychiatrist. She said that my therapist is known in the psychiatric community for being someone who doesn’t actually assess you for gender dysphoria, who just gives everybody the referrals… She’s known for giving the rubber stamp on everyone and she doesn’t do any discriminatory, like, differential diagnosis… Maybe two years of therapy, it’s a bit excessive and harms trans people… And three sessions of an hour-long, like, [gender-]affirmation therapy, is also harming people that are just gender non-conforming. [T]here’s a middle ground… I think if I had actual therapy, I would have worked out that I was a lesbian before I transitioned. (Participant #10, female, age 29).
	I started testosterone at age 15… I was presenting as very, very masculine, and I was in the system… the people I was working with [group home staff] had brought in a gender identity specialist, and they asked me: “This is transgender, are you this?” And I’d never heard of it before… I think I definitely also saw it as a portal to escape, like, my girlhood, AFAB childhood… Blockers, testosterone, wearing a binder, changing my name, changing my name legally, going on the list for top surgery when I was 17, which I did not get… There was a formula. I didn’t ask for it, it was kind of a given… I wasn’t viewing it as this is something I’m doing for the rest of my life, this is something that’s going to impact my social life, my romantic life, my, you know, the way I see myself, the way I like myself, my body, et cetera… I would probably have held off the testosterone… I had a pretty male socializing for a long time, and I don’t think I can undo all of that, and I think that’s why I’m more masculine now. (Participant #13, transmasculine nonbinary, age 25).
***Support for Autonomy and Individualized Care Pathways***	
	The biggest advice [to providers] above everything is listen to people. And I think that goes beyond being trans. Listen to people. That if they tell you something is happening, it’s because something’s happening. Why would you doubt. . . When it comes to, for example, transitioning, as I mentioned, I had to fabricate stories to seem “trans enough” for the doctors. But beyond that, beyond listening, it’s also. . . Putting your thoughts about identity at the door. (Participant #4, nonbinary transmasculine, age 24).
	I guess you shouldn’t look at patients seeking gender care as like, someone seeking like antibiotics for a bad cold or something like that. [Providers] need to look at it as someone who’s seeking a way to sort of comfortably find a way to express themselves and to sort of make peace with who they are. But that means that it’s going to vary from person to person what they need and what they’re comfortable with (Participant #17, gender-fluid male, age 32).
	Now when I look back on it, yeah, I was probably led [to transition]. Because I was in a spot where I… I really needed an out. And [transition] did get me out… I always knew that I was some kind of queer… Been attracted to women or people who have vaginas, and that. I knew that I wasn’t straight… I grew in very homophobic house. And so, I think that also may have played into it [identifying as a trans man and transitioning]… I think probably mental health counseling to deal with my trauma, probably would have prevented me from going through the transition. (Participant #9, undecided, age 37).
	[To a theoretical therapist:] ‘This is my life story, what would benefit me? Where should I go from here?’… And I think that with neutral therapy it would have been an exploration and a discussion around why… As a girl, actually questioning the gender stereotypes that I held and that black and white thinking of. . . “boys get to date girls, girls can’t… Well, why?” (Participant #10, female, age 29).
	I feel like I should have never really went down this path… I feel like, if I would have been supported to say that there’s nothing wrong with being gay. There’s nothing wrong with being a female who is interested in other females… I feel like maybe [my transition] wouldn’t have happened, if you know what I mean?… I think I just had this shame for being female… So like just growing up and kind of getting in a better environment where I could have more the space to, look at myself. And have the space to kind of go for what I want, outside like parents’ influence or other people’s influence (Participant #14, female, age 23).
	Healthcare practitioners need to listen to their patients. Need to take into account everyone’s unique circumstances. And particularly with trans care I think it’s really important that they [clinicians] not be kind of married to this specific idea of the transgender pathway, or trajectory… (Participant #16, nonbinary, age 30).

### Binary gender assessments and linear transitions

The vast majority of participants accessed medical/surgical transition through the gender-affirming model of care (*SOC-7*), with participants’ descriptions of assessments revealing little standardization of practice across different clinics/regions of Canada. Many also engaged in talk therapy with a mental health professional, such as a psychologist or psychiatrist, in order to receive a letter to start hormones because it was required by their prescribing physicians or by provincial healthcare policy at the time. The assessment process ranged from between one month to over one year. Only a few participants reported being provided hormones after one or two visits at what participants often themselves called “informed consent” clinics.

Participants observed that assessment practices, together with healthcare policies, ultimately put measures in place to prevent regret and detransition, which was thought to extend the time to access the desired interventions. As one participant put it:

The province [public insurance] puts up all these barriers being, like, “But are you sure? Are you mentally fit? Do you know that this is what you want? Are you going to change your mind?” … The way that the nurse actually described it to me was that they are just following the provincial rules… I contacted the trans [clinic], which, if you get any gender-affirming surgeries or hormones or anything in [province], you have to go through the [trans clinic]… And then if you say: “No, I don’t want to wait on that waitlist, I want to do it privately”, [psychologist name is] the only one in the entire province who is certified to do the [assessment and] referral (Participant # 11, nonbinary, age 26).

As a result of these structured, stepwise assessment procedures outlined by the *SOC-7*, and long waitlists, participants’ gender transitions largely conformed to a linear pathway, such as starting hormones prior to surgery—regardless of their own individual desires. In turn, many participants felt constrained or felt there was external pressure on their transition. Some pursued hormonal interventions that they later came to question or regret because this was required to access surgery.

For many, their gender transition was lengthy and drawn-out. One participant who had transitioned approximately twelve years prior to detransitioning had a therapist as well as fulfilled the *SOC* (version 6) criteria which included living for one-year in “your chosen gender” prior to initiating hormones, and then waited for the province to insure surgery:

At that time, you still had what’s called the *Harry Benjamin Standards* [WPATH], you had to be living as male, in your chosen gender for a year before they [providers] would give you hormones. So, I came out, I changed my name. I was binding, dressing as male, presenting as male at work and to my friends and family. And then I had a hysterectomy, and then I started testosterone right after that. And then, took testosterone for a number of years. Then surgery became covered in [province]. And then I had [chest] surgery and [genital] surgery at the same time (Participant #9, undecided (she/her), age 37).

This participant experienced what was called “the real life test” prior to initiating hormones, which was a practice in place at the time she transitioned. Her medical/surgical transition process was drawn out for years before she ultimately detransitioned following shifts in gender identity and genital surgery complications.

The process of being assessed for medical/surgical transition was not experienced as “gender-affirming” by any of the participants. They felt this approach was applied inflexibly and that it was less about offering support and ensuring they had good outcomes, and more about enforcing binary transgender identities and placing limitations on treatment options.

In my early 20s I started to look at what is gender, and I’ve never identified as a man, but a lot of people put that on top of me… I pursued top surgery… I got switched to a different psychiatrist who’s awful [and who said] “You can’t be trans because you don’t want to be on testosterone.” (Participant #12, gender-fluid, age 29).

Participants frequently felt a need to prove themselves eligible for care and “trans enough”, by jumping through pre-determined “hoops” set by healthcare policies and providers. That said, many participants still felt satisfied with the overall transition process and decided to discontinue gender-affirming hormones because they were content and/or because they shifted from binary transgender to nonbinary.

### Dissatisfaction with “informed consent” practices and decisional regret

Despite informed consent clinics being described as scarce and hard to access, participants frequently endorsed the ICM, including one participant who explained that an “informed consent” provider “was the only doctor that I felt comfortable enough with to tell her that I had detransitioned.” (Participant #7, cis woman, age 29). However, participants largely described providers’ informed consent practices as being inconsistent with principles of informed consent such as having robust discussions about a range of treatment options and their known risks/benefits. Reflecting back years after initiating these treatments, many participants did not feel adequately equipped to make informed decisions. When decisional regret emerged as a theme, it was frequently described alongside dissatisfaction with care providers’ practices such as feeling under-informed about interventions. As reported elsewhere, one-third of this sample expressed regret or complicated, ambivalent feelings about their decision to transition [14].

Participants often reported unexpected, negative health issues they attributed to hormone-related treatments, or of being disappointed with the individual, embodied effects. For instance, the only participant who reported puberty blockade (Lupron) followed by testosterone shared that:

I think there should be more actual medical studies or medical awareness of what stopping testosterone means [after blockers], because I developed… a bunch of hormone disorders and I’d also never actually gotten my assigned female at birth puberty before [stopping testosterone]… I got my first menstruation at 19, and my body’s basically a disaster (Participant #13, transmasculine nonbinary, age 25).

As shown above, they felt they may have suffered negative health consequences, and that more research is needed to improve medical knowledge of this specific treatment pathway. Others recommended more transparent discussions to explore the possibility that the effects of medical treatments may not align with their individual embodiment goals and could thus be disappointing. For instance, one participant stated: “Nobody said ‘Hey, you go on testosterone you still might not look like a guy.’ That is possible, but no one tells you that.” (Participant #27, uncertain identity, age 25). Many explained that providers presumed they were already sufficiently knowledgeable, and some participants recalled receiving lengthy packets of information regarding prospective interventions with little verification that they had understood relative risks/benefits of the treatments.

Both participants who initiated medical transition at age 15 expressed regret and dissatisfaction with hormonal/surgical interventions’ lasting impact on their bodies, gender socialization, and gender expression.

My gender expression is not where I’d like it to be, but that’s for a future thing. I would definitely like to express myself more in a feminine way… I mean, they gave me hormones pretty much after just one appointment… I wasn’t really even like asked about my gender, really, during the appointment. I wasn’t asked about why I wanted to be a boy or challenged with that. I was just sort of screened for general mental illness—symptoms of hallucinations, or aggressive behavior, and stuff like that. I didn’t receive really any in-depth questioning. It was like: “Okay you think you’re a boy and that’s cool. We can do that for you.” I feel like I deserved better care in a sense… [That] they actually took the time to figure out … where those thoughts with wanting to be a boy were, why I wanted to go on testosterone? I was so young [age 15] at the time. Right? Like… I was just thinking, I want to get away from being a girl… I feel like if I would have been supported to say that there’s nothing wrong… with being a female who is interested in other females… this [transition] would not have happened. (Participant #14, female, age 23).

Like the above account, a few other participants felt that medical transition presented them with an opportunity to “escape” or to “get away from being a girl” without fully appreciating a deeper significance of this desire. As young adults with identities that are different today than when they first pursued medical transition, they described internal and external regrets, and felt their providers’ consenting procedures were not adapted to meet their age-specific developmental needs.

Several participants reflected there was a lack of transparency and communication with regard to treatment decisions in the context of mental health, with some speculating that an in-depth discussion about sexual and gender diversity and/or a comprehensive assessment may have identified factors contributing to their feelings of gender dysphoria—information thought to be important to fully exercise their autonomy and make informed treatment decisions. Some participants felt that their trans identity and/or pursuit of medical transition may have been partially motivated by ego-dystonic homosexuality, neurodivergence (e.g., autism), or a past trauma that was, at the time, not transparent to themselves nor proactively identified by providers.

When I detransitioned, it was about six years ago… I had some space to process some of the reasons why I thought I had to identify away from womanhood in the first place. And I know it’s not everyone’s experience, but for me, I realized that a lot of it was tied up with like other things like trauma, instead of my actual gender identity… Or like when I was growing up, I had undiagnosed autism, which I actually think does relate to my gender identity (Participant #7, cis woman, age 29).

These participants came to understand a range of other factors as intersecting with their embodied experience of gender/gender dysphoria only after detransitioning—signaling a missed opportunity during the care-seeking process. Another participant who, after detransitioning came to understand her past body-related distress through a lens of complex mental illness, questioned the context under which she provided consent, sharing that:

When I signed the consent forms in [month] 2017 for hormones, I was actually in the psychiatric hospital. So, I got a day pass from inpatient. I was sectioned. They did a *Form 1* [psychiatric care admission under the *Mental Health Act*] and I was held for being a danger to myself, a high risk for suicide or self-harm. But they gave me a day pass so that I could go to see the endocrinologist and sign informed consent. And the question that I hold now is, “how is that informed consent?” If I’m a danger to myself and I’m not allowed to go home without the police coming and bringing me back to the hospital, am I really mentally competent to understand the long-term effects of hormone therapy?… (Participant #10, female, age 29).

This participant questioned how she was able to provide consent for treatment during “high risk” periods of her life, and also expressed regret with regard to all completed interventions (testosterone, chest masculinization surgery, oophorectomy). Others observed that some providers—in their desire to reduce service access barriers and offer support and affirmation to trans people—may not be completing thorough informed consent procedures. Another participant reflected the sentiment that there is a “fine balance between gatekeeping and doing due diligence” (Participant #15, nonbinary, age 29). A theme thus emerged of recommending a “middle ground” or “diligent” practice to mitigate the risks of delaying gender care, while providing care-seeking individuals time and space to clarify their gender identity, identify individualized mental healthcare needs, and whether they might be experiencing what some described as “internalized homophobia” or generalized societal homophobia which could impact feelings about their gender.

### Supporting autonomy and individualized care pathways

Participants supported autonomy in patient decision-making and the ICM in particular. The need for an individualized approach to care, inclusive of mental healthcare and social supports, was frequently discussed. A majority of participants ultimately felt that medical/surgical transition was the right choice for them at the time, centered themselves as active agents involved in their own decision-making, and reported positive feelings about their gender transition. However, some also reflected on how they might have felt better supported in their decision-making process, particularly those who reported complex mental health challenges such as BPD, PTSD, or bipolar disorder. For instance, one participant shared that:

I definitely do not think everyone with BPD who transitions is going to detransition. I do not think that at all. But for some people, I think that would actually be something for professionals to kind of look into… Like even if it involved doing some sort of group therapy just to kind of help build up your sense of self. I think that would be really–something really important… because [gender-affirming] hormones really do take a lot of effects on you. (Participant #20, female, age 25)

Although she felt that transitioning was the right decision, and reported overall good relationships with her care providers, she also felt a more individualized care approach with therapeutic supports was warranted in her case, specifically due to her experience of living with BPD.

By contrast, a minority of participants felt medical/surgical transition was the wrong pathway and, at the time of interview, they shared that they wished they been provided with more supports and in-depth, “neutral” therapy to examine how their feelings about their sexual orientation or being gender non-conforming may be impacting their gender dysphoria. Some felt they would have benefitted from a more comprehensive assessment in order to be better prepared for treatment decision-making, because in hindsight, they realized medical/surgical interventions did not address their needs. Reflecting back, these participants felt that having time and space to explore their life histories and possible connections with gender dysphoria, gender identity development, and embodiment desires would be valuable for care-seeking individuals. For these participants, their narratives contained language indicative of feelings of suggestibility and the notion that social oppression (e.g., homophobia/heterosexism; sexism) may have shaped their life experiences and pursuit of medical/surgical interventions. Finally, it was recommended that providers take a “neutral” stance, listen, offer space for identity exploration, and identify individualized needs:

[Providers should] really just encourage the clients, or the patients, as individuals to speak on their own experiences and their own perceptions and to encourage that, and…not project their views on gender, or their views, on this person’s gender (Participant #13, nonbinary transmasculine, age 25).

## Discussion

We interviewed individuals who shifted/reversed their gender transitions about their care experiences and perspectives on practice. The overwhelming majority of participants had initiated medical/surgical transition through the gender-affirming model of care, with many also reporting having a therapist or mental healthcare provider such as a psychologist or psychiatrist. To improve the care experiences of people seeking gender care, we recommend that providers: (1) expand the “informed” in informed consent; and (2) offer care that embraces individualized gender transition pathways that is sensitive to the distinctive needs of trans, nonbinary, and gender non-conforming sexual minorities.

We found that the gender-affirming model (e.g. *SOC-7*) was not actually experienced as gender-affirming because it favoured binary transgender identities. This could be due to 60% of our sample re-identifying as nonbinary after beginning their transition having initially identified as binary trans (e.g., trans man or trans woman). Although participants understood assessments were in place to ensure they were not “making a mistake”, this approach seemed to reify a lengthy transition process and long waits for services, followed by an informed consent process within the clinic setting that was perceived to be rushed or insufficient. Thus, the current constrained system comprising long wait lists for gender care services may inhibit exploration, self-discovery, and achieving fully informed consent. These findings extend other authors’ conclusions that poor service access and long wait times for an assessment in Canada are distressing [27,28,49], that informed consent procedures in gender care are often insufficient [33,38], and that assessments are unlikely to reliably predict whose gender/sexual identity will change over time [37,50]. Healthcare delivered within neoliberal environments is under tremendous pressure to cost-contain, which translates into care delivered to meet the system’s needs—to the detriment of quality patient care [51,52]. We recommend that healthcare policy decision-makers strive to reduce wait times, while also developing publicly-funded social and mental health support programs that individuals can access while waiting for, and accessing, care services.

Participants overwhelmingly endorsed individualized care options and a more robust informed consent process—with variation in how people thought this ought to be done in practice (e.g., stronger emphasis on differential diagnosis within a therapeutically collaborative environment, versus removing gender dysphoria diagnosis and mental health assessment requirements entirely). There was a divergence in perspectives about discussing with providers potential links between sexual orientation and gender dysphoria. Although there is no current research substantiating a causal relationship between ego-dystonic homosexuality and gender dysphoria/trans identity, studies of largely AFAB detransitioned women reported similar findings [13,15]. This warrants further empirical examination divorced from *a priori* beliefs that gender transition is entirely driven by homophobia/biphobia/heterosexism and other social factors, as well as the *a priori* assumption that gender transition is never socially-mediated.

Now in their early to mid-twenties, the two participants who began medical transition at age fifteen expressed that, at the time, they did not fully understand the future implications of this decision—emerging phenomena also found in other studies of detransition among young adults [13,15]. Of these two participants, one reported family support and the other described a “rough” childhood and was living in a group home. Both lived as trans men for several years (5–10 years) before detransitioning due to feeling uncomfortable in a male social role and reported a change in their identity. Vrouenraets et al. assessed Dutch trans youth’s (mean age 14.71) medical decision-making competence to initiate puberty suppression, finding that 93.2% of the AFAB participants were competent to consent [53]. However, these authors’ describe an approach to achieving informed consent via monthly sessions with a mental health care provider over a period of ~6 months, which diverged from what the participants in our sample recalled of their own care as adolescents. Another qualitative study of 20 detrans youth (aged 16–25; 95% AFAB) found that some participants expressed decisional regret and that they lacked sufficient support when making treatment decisions [21]. This is consistent with those who reported regret in our study. While respecting TGD self-determination, given these findings we recommend robustly examining adolescent patients’ 10-year outcomes on a larger scale in terms of identity development and satisfaction with interventions, as these findings seem to confirm some providers’ concerns that some youth may lack insight into the possibility for an evolving gender identity [54]. Identity shifts should be studied further to improve care providers’ understanding of LGBTQ2S+ lives and to better inform care-seekers about associated implications for satisfaction with medical/surgical outcomes, which complements other research [50,55–57].

Centering autonomy and bolstering informed consent in gender care may limit decisional regret. The subset of participants who expressed regret often felt that they lacked sufficient information or support to make the right treatment decision at the time of transitioning, yet they still endorsed autonomy. Reflecting back, they felt they lacked insights such as the extent to which their sexual orientation, mental illness, or neurodivergence may have intersected with dysphoria or desire to transition and many said they would have benefitted from “neutral” therapy. Some might interpret these individuals’ regret to misdiagnosis or to never being “truly” trans. Yet, some currently affirmed a TGD identity. And as we presented elsewhere, several other participants in this sample who re-identified with their assigned sex (e.g., do not identify as TGD) feel positive about their experience of medical transition and their current embodiment after transition [14]. Taken together, decisional regret may be more about lacking information about the relative risks/benefits/alternatives of various treatment options and desiring of more clarity about themselves than having too much autonomy. This is consistent with a systematic review of 12 studies that identified a clear relationship between decisional regret and insufficient information provided to women who had cancer-related mastectomy [58]. Two prior surveys also found detransitioners felt they were under-informed about the risks and benefits of medical transition with a majority in both studies reporting regret [13,15].

Our results do not seem to lend support for therapy that is not gender-affirming, nor for therapy for an undefined length of time prior to initiating medical/surgical care. Instead, participants largely desired a “neutral” approach to care that is discerning of individual needs and provides the *option* to explore their sexuality/gender/gender dysphoria, to fully discuss and consider a range of interventions and therapies, and to receive concurrent mental health supports. Participants also recommended that providers avoid projecting their own views of gender. These findings indicate support for practices outlined by the updated *SOC-8* adolescent and adult assessment chapters which promote gender identity exploration without favouring any particular identity outcome while recommending individualized care [30].

## Limitations and conclusion

Our study has several strengths and limitations. This was the first national, qualitative study to explore detransition/retransition among a heterogenous sample of 28 adults. We employed an exploratory, qualitative study design to collect in-depth, narrative interview data. Our study used similar online recruitment strategies as past studies of detransitioners—including targeting gender care clinicians, TGD-serving and detransitioning groups, and general social media study promotion—however we also created tailored recruitment flyers to reach AMAB/transfeminine spectrum and BIPOC communities who have been underrepresented in this area of research. In comparison to the past surveys, the present study has a higher portion of participants who reported a current TGD identity (75%), trans women and AMAB (36%), and BIPOC individuals (18%). Still, given the small sample size and non-probability sampling, results should be taken as exploratory and lacking generalizability.

Likely due to healthcare policies in Canada, a majority of participants experienced the gender-affirming model of care, thus our analysis focused largely on this model. To our knowledge, none of the participants directly experienced the gender exploratory therapy model, however a majority of participants discussed having a regular mental health provider before and during their transitions. We did not investigate what modality of talk therapy participants received. It is plausible that our findings would differ if more participants had experienced the ICM or the gender exploratory therapy model. Finally, given that participants were recalling events that spanned great lengths of time, recall bias likely limited participants’ recollection of their feelings about their gender transition, gender dysphoria, and experiences with care providers. The extent to which participants were satisfied or dissatisfied with the somatic outcomes of medical/surgical interventions may have also coloured their recommendations for care delivery and any feelings of decisional regret. Prospective, longitudinal research is needed to rigorously examine the extent to which this population’s gender/sexual identities and embodiment desires evolve over time, satisfaction with care and treatments, and their overall perspectives about their gender transitions/detransitions/retransitions at different timepoints across the life course.
